# Reactive Sintering of Ground Tire Rubber (GTR) Modified by a Trans-Polyoctenamer Rubber and Curing Additives

**DOI:** 10.3390/polym12123018

**Published:** 2020-12-17

**Authors:** Łukasz Zedler, Daria Kowalkowska-Zedler, Xavier Colom, Javier Cañavate, Mohammad Reza Saeb, Krzysztof Formela

**Affiliations:** 1 Department of Polymer Technology, Faculty of Chemistry, Gdańsk University of Technology, Gabriela, Narutowicza 11/12, 80–233 Gdańsk, Poland; 2 Department of Inorganic Chemistry, Faculty of Chemistry, Gdańsk University of Technology, Gabriela, Narutowicza 11/12, 80–233 Gdańsk, Poland; daria.zedler@pg.edu.pl; 3 Department of Chemical Engineering, Universitat Politècnica de Catalunya Barcelona Tech, Carrer de Colom, 1, 08222 Terrassa, Barcelona, Spain; xavier.colom@upc.edu (X.C.); francisco.javier.canavate@upc.edu (J.C.); 4 Center of Excellence in Electrochemistry, School of Chemistry, College of Science, University of Tehran, Tehran 11155-4563, Iran; mrsaeb2008@gmail.com

**Keywords:** ground tire rubber, modification, compatibilization, waste management, reclaiming, recycling

## Abstract

The proposed method of ground tire rubber (GTR) utilization involves the application of trans-polyoctenamer rubber (TOR), a commercially available waste rubber modifier. The idea was to investigate the influence of various curing additives (sulfur, N-cyclohexyl-2-benzothiazole sulfenamide (CBS), dibenzothiazole disulfide (MBTS) and di-(2-ethyl)hexylphosphorylpolysulfide (SDT)) on curing characteristics, physico-mechanical, thermal, acoustic properties as well as the morphology of modified GTR, in order to evaluate the possibility of reclaiming GTR and the co-cross-linking between applied components. The results showed that the presence of the modifier without the addition of curing additives hinders the physico-mechanical properties of revulcanized GTR. The addition of SDT, CBS, MBTS and sulfur change the melting kinetics of TOR, indicating partial degradation and/or co-cross-linking between components. In the studied conditions, the best mechanical properties were obtained by the samples cured with sulfur. The morphology analysis, combined with the physico-mechanical results, indicated that when the surface of the GTR is more developed, obtained by the addition of TOR, the properties of the GTR improve.

## 1. Introduction

The growing demand for tires, due to increasing consumerism and logistics solutions, contributes to the generation of an increase in waste rubber, which is structurally challenging to recycle [1,2]. Researches, and activities of national governments, have been undertaken for years in order to find appropriate solutions to treat this problematic material which presents a threat to the environment and human health [3]. Consequently, developing an environmentally friendly process for the disposal of waste tires is one of the most important tasks of the 21st century for state authorities, manufacturing companies, and scientists. 

At present, the most applied method for waste rubber utilization is energy recovery [4]. The caloric value of a tire has been estimated at 32 MJ/kg, which is comparable with other types of fuels, such as coal, which has the caloric value at 26 MJ/kg [5]. However, compared to the energy required to produce one tire (87–115 MJ/kg) [3] the possible output of energy recovery from an end-of-life tire is quite low and a claim for a process that, instead of wasting generated energy, would allow the recycling and the preparation of products with satisfactory properties, suitable for industrial applications, has arisen.

A high number of published studies, covering the subject of waste rubber management, focus on specific recycling that uses ground tire rubber (GTR) as input material. The rubber in this form can be processed via methods that generate shear forces, transforming part of the material into sol by the scission of cross-links and main chains [6,7]. The product in this state can be more easily processed and vulcanized than raw GTR [8,9]. Its structure mostly consists of a sol fraction covering untouched rubber particles [10,11]. The ratio of rubber to a soluble part depends on the reclaiming method [12], which usually has a mechanical, physical, chemical, biological, or combined background. Such treatment produces not only scission of the cross-links and main chains, which translates to an improvement of flowability, but also oxidization of the surface, resulting in the appearance of hydroxyl groups [13]. This phenomenon can be used for the compatibilization of GTR with other matrices, such as polymers, bitumens, or concretes. 

The application of GTR in a thermoplastic matrix is a well-known strategy among which blending, compatibilization via surface modification [14,15], and the use of functionalized block copolymers can be applied [16]. The idea of using GTR in thermoplastics has a very good economic and ecological basis, resulting from the assumption that every treatment of GTR should be cost-effective, while the final product is expected to have satisfactory properties. The fact that thermoplastics are one of the more economically viable plastics also encourages their use in GTR management technologies. However, the application of thermoplastics with GTR also has its limitations. As mentioned before, the plastic needs to be modified, and this results in additional costs connected to the treatment process. Moreover, increasing the content of GTR in many cases causes a deterioration of mechanical properties. In such a case, it is preferable to use a material with a good affinity for GTR. 

Among commercially available products a trans-polyoctenamer rubber (TOR) can be found under the tradename Vestenamer 8012. It is a semicrystalline polyolefin additive with a high proportion of trans double-bonds, which is dedicated to waste rubber recycling. The additive plays two important roles in terms of GTR recycling. First, it acts as a plasticizer that helps the processing of the cross-linked waste rubber. Second, due to the presence of unsaturated bonds, it takes part in the vulcanization process that facilitates the cross-linking of the GTR and also the co-cross-linking between GTR and TOR [17].

The available literature reports and patents mainly concern the application of TOR in asphalt mixtures, to which GTR is added [18,19,20]. Arti et al. [21] investigated the influence of three types of peptizers: Vestenamer 8012, Aktiplast 8, and Rhenosin 145 on the mastication of natural rubber/butadiene rubber blends, proving that Vestenamer 8012 can significantly increase mechanical properties while optimizing the mastication process. 

Wang et al. [22] prepared blends based on recycled PE (rPE) and GTR with GTR contents ranging from 0% up to 90%. Among the samples, several were selected and then modified by adding the commercially available copolymers Vestenamer 8012 and Engage 8180. The materials were melt blended via extrusion and injection molded to obtain specimens for tests. The mechanical properties of rPE/GTR (10/90) compatibilized with 9 phr of the cited copolymers showed significant superiority of Vestenamer 8012 (tensile strength approx. 2.1 MPa and elongation at break approx. 70%) over Engage 8180 (tensile strength approx. 0.9 MPa and elongation at break approx. 72.5%). The SEM analysis indicated that the addition of the mentioned copolymers had a positive effect on the morphology of the studied materials. 

Herrmann et al. [23] modified GTR with mercaptobenzothiazole (MBT) and sulfur by compounding in a fluid mixer. The same modification was carried out melting GTR blended with TOR. Such treated waste rubber was mixed with a natural rubber/styrene-butadiene rubber blend (in ratio 70/30), including components typically used for truck treads, and then vulcanized at 155 °C. The physico-mechanical analysis indicated that the incorporation of modified GTR (regardless of the type of modification) did not improve the bonding between the matrix and the GTR particles (unmodified GTR—14.9 MPa, 426.6%; GTR/TOR—14.9 MPa, 436.1%; GTR/TOR/MBT—15.6 MPa, 430.0%; GTR/TOR/MBT/Sulfur—16.1 MPa; 421.8%). The authors also introduced modified GTR in a natural rubber matrix. The addition of the modified GTR caused a decrease in parameters, and any expected differences between the types of modified GTR (GTR, GTR/TOR, GTR/TOR/MBT, or GTR/TOR/MBT/sulfur) in the analyzed composition were possibly masked by the much stronger matrix of natural rubber.

All of the published studies used TOR as a compatibilizer between GTR and various matrices. However, according to our knowledge, there is no published report about the influence of curing additives on cross-linking and co-cross-linking of TOR and GTR. 

In the presented work, GTR was mechano-chemically reclaimed in the presence of common curing additives used in the rubber industry: accelerators and two types of sulfur. The choice of using these systems without applying any other typical compounds generally included in the vulcanization, responds to the need for achieving a process to recycle GTR that must be as economically viable as possible. Moreover, it has been proven that, when preparing mixtures using waste rubber, there is a migration of unreacted components, including accelerators or plasticizers [24,25]. Therefore it is theoretically possible to carry out reclaiming and revulcanization processes using, for example, sulfur alone. The modified reclaimed rubbers have undergone a reactive sintering process, and the obtained revulcanizates were characterized by curing and swelling characteristics, tensile tests, hardness, density, Fourier-transform infrared spectroscopy, thermogravimetric analysis, scanning electron microscopy, acoustic properties, and differential scanning calorimetry. 

## 2. Experimental

### 2.1. Materials 

**Figure 1:**
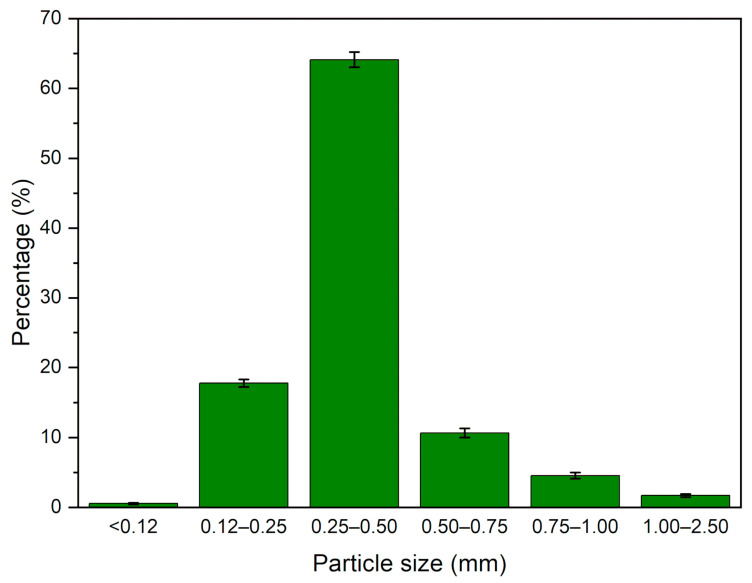
Ground tire rubber (GTR) particle size distribution.

Ground tire rubber (GTR) was received from Grupa Recykl S.A. (Śrem, Poland). The particle size distribution of the used GTR is presented in Figure 1. The material was obtained by grinding at the ambient temperature of end-of-life tires (mix of passenger car tires and truck tires). 

Vestenamer^®^ 8012 was provided by Evonik Resource Efficiency GmbH (Essen, Germany). According to the manufacturer, a small addition to a compound improves the mixing and processing of a sample. Moreover, it enhances the dispersion of difficult polymer blends, reducing the viscosity of the compound, and acts as a compatibilizer between different rubber types. Vestenamer 8012 is a trans-polyoctenamer rubber (TOR) whose characteristics are presented in Table 1. 

Sulfur was provided by Standard Sp. z o.o. (Poland, Lublin), while N-cyclohexyl-2-benzothiazole sulfenamide (CBS), dibenzothiazole disulfide (80%) dispersed in elastomer binder (20%) (Rhenogran^®^ MBTS-80), di-(2-ethyl)hexylphosphorylpolysulfide (50%) dispersed in elastomer binder (50%) (Rhenogran^®^ SDT-50) and sulfur (80%) dispersed in elastomer binder (20%) produced by Lanxess (Cologne, Germany).

### 2.2. Sample Preparation

Mechano-chemical reclaiming was conducted at ambient temperature utilizing the two-roll mills model 14201/P2 from Buzuluk (Komárov, Czech Republic). The whole process time was set to 10 min and the application order and time of used components were as follows: GTR (start), TOR—Vestenamer 8012 (after 1 min), and curing additives (after 7 min). The following two-roll mills settings were used: ambient temperature, friction equaled 1.08 and the gap width varied between 0.2 and 3 mm. 

The composition of the obtained samples is presented in Table 2. The variable amount of additives in the presented composition results from the need to recalculate them so that the same amount of active compound (3 phr—parts per hundred rubber) is added to each sample. After the mastication samples were submitted to the revulcanization process by forming them into sheets with 2 mm thickness and cured in an electrically heated press (PH-90, Nysa, Poland) at 180 °C under the pressure of 4.9 MPa according to the optimum cure time determined as stated in ISO 3417 standard. To make it simple, special coding of tested samples was used according to GTR/TOR^X^ where GTR is a ground tire rubber, TOR stands for Vestenamer 8012 and X stands for applied curing agent/accelerator. 

### 2.3. Measurements

The vulcanization process of prepared samples was investigated via a Monsanto R100S rheometer with an oscillating rotor (Monsanto Company, St. Louis, MO, USA) in accordance with ISO 3417. In order to determine the cross-linking rate, the cure rate index (CRI) was calculated according to Formula (1) [26]:

(1) $$\mathrm{CRI}=\frac{100}{t_{90}-t_{2}}$$

where: t_90_ is the optimum vulcanization time, min and t_2_ is the scorch time, min.

Determination of the R_300_ parameter led to investigation of the aging resistance of prepared samples at a raised temperature. R_300_ is calculated from the time at which torque reaches the maximum value (M_max._) and it describes the percentage of reversion degree after a period of 300 s [27]. It was calculated according to the Formula (2):

(2) $$R_{300}=\frac{M_{\max}-M_{300}}{M_{\max}}\times 100\%$$

where M_max._ is the maximum torque, dNm and M_300s_ is the torque 300 s after the occurrence of the maximum torque, dNm.

The tensile strength and elongation at break were estimated in accordance with ISO 37. Tensile tests were carried out on the Zwick Z020 machine (Ulm, Germany) at a constant speed of 500 mm/min. Direct extension measurements were conducted periodically using an extensometer with sensor arms. The reported results stem from five measurements for each sample. Shore hardness type A was assessed using the Zwick 3130 durometer (Ulm, Germany) according to ISO 7619-1.

Based on the Archimedes method, explained in ISO 1183, the density of the vulcanized samples was determined. Measurements were carried out at room temperature in a methanol medium, without exception. 

The swelling degree of vulcanized samples (0.2 g) was estimated via a swelling test carried out in toluene at room temperature. The swelling degree was calculated according to Equation (3):

(3) $$Q=\frac{m_{t}-m_{0}}{m_{0}}\times 100\%$$

where Q is the swelling degree, %; m_t_ is the mass of the sample swollen after time t, g; and m_0_ is an initial mass of the sample, g.

Sol fraction was calculated in accordance with formula (4):

(4) $$\mathrm{Sol}\;\mathrm{fraction}=\frac{W_{1}-W_{2}}{W_{1}}\times 100\%$$

where: W_1_ is a mass of the vulcanized sample before swelling, g; and W_2_ is the mass of the vulcanized sample after extraction, g.

Acoustic properties were measured using a two-microphone impedance tube Brüel and Kjaer type 4206 (Darmstadt, Germany) in the frequency range 100–6500 Hz, according to ISO 10534-2, which describes the test method for impedance and absorption of acoustical materials using a tube, two microphones and a digital frequency analysis system. The sound absorption coefficient (α) is defined as the ratio of energy absorbed by the sample (E_a_) to the total incident energy acting on the sample energy (E_i_) on a sample, as presented in equal (5):

(5) $$\alpha =\frac{E_{a}}{E_{i}}=1-{\left(\frac{n-1}{n+1}\right)}^{2}$$

where the parameter n parameter is correlated to the ratio between the measured maximum (p_max_) and the minimum (p_min_) sound pressure inside the tube, as shown in the Equation (6):

(6) $$n=\frac{p_{max}}{p_{min}}$$

The morphology of GTR and reclaimed GTR was characterized by a JEOL 5610 scanning electron microscope (Tokyo, Japan). Before the analysis, samples were coated with a thin layer of gold. 

The FTIR spectra were measured in the range of 4000 to 650 cm^−1^ with a Nicolet iS50 FT-IR spectrometer (Waltham, MA, USA) equipped with the Specac Quest single-reflection diamond attenuated total reflectance (ATR) accessory. Spectral analysis was controlled by the OMNIC software package version 9.8.372.

The thermal analysis of GTR and modified GTR was performed using TGA (thermogravimetric analysis) model TG 209F3 from the Netzsch Group (Selb, Germany). Samples were weighed to approx. 10 mg and placed in a corundum pan. The study was conducted in an inert gas atmosphere containing nitrogen (flow rate of 20 mL/min) in the range from 35 to 820 °C with a heating ramp of 20 °C/min.

The thermal behavior and crystallization of the samples were measured by differential scanning calorimetry (DSC). The measurement was carried out on a DSC 204 F1 Phoenix apparatus (Netzsch Group, Selb, Germany). Samples of approx. 9 to 10 mg were placed in an aluminum pan and heated from 20 °C up to 250 °C at the rate of 10 °C/min. The cooling was carried out from 250 to −80 °C at the rate of 10 °C/min; subsequently, materials were heated a 2nd time from −80 to 250 °C at the rate of 10 °C/min. 

## 3. Results and Discussion

### 3.1. Curing Characteristics

**Figure 2:**
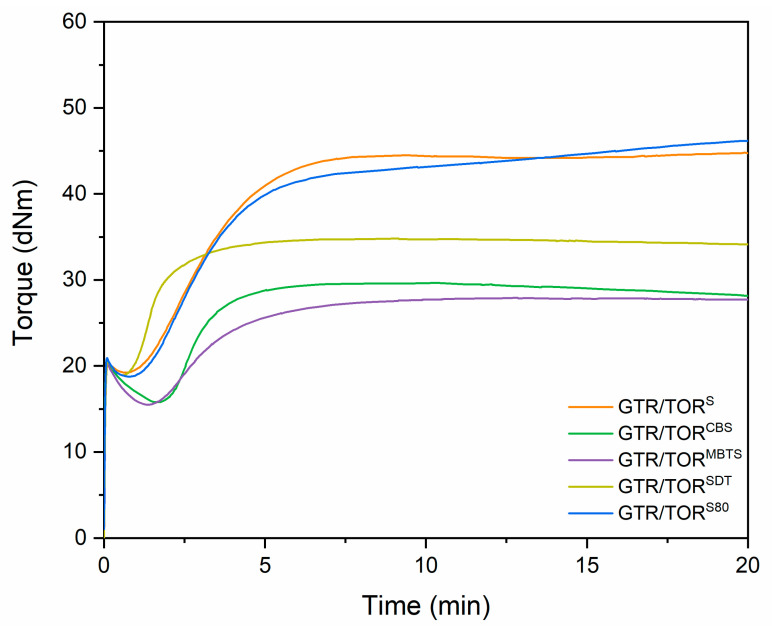
Curing curves of studied samples performed at 180 °C.

The effect of used additives on the curing characteristics of GTR modified with TOR is presented in Figure 2 and summarized in Table 3. As can be seen for samples GTR and GTR/TOR the curing data were not recorded. This is due to the fact that those samples do not contain any cross-linking supporting components. It is well-known [28,29] that during the compounding of GTR, the shear forces acting on the material not only cause the scission of polymeric chains and/or cross-links but also support migration of unreacted components (for example sulfur or accelerators) and carbon black from the ground tire rubber to another polymeric matrix or within the waste material itself. This phenomenon could influence the curing of GTR and GTR/TOR and the results in a typical curing curve course. However, the amount of released components is too small, being impossible to observe the differences in curing behavior by an oscillating disk rheometer. For further analysis of those samples, the curing time was set to 5 min. For other studied materials, the curing took place and the most crucial parameters were measured and listed in Table 3.

Minimal torque (M_min._) is a value that gives the first insight into the processing of a sample. The lower the M_min._, the better are the processing properties of the sample. In this case, the value depends on the type of applied agent and decreases as follows: 19.2 dNm (S), 18.9 dNm (SDT), 18.7 dNm (S80), 15.8 dNm (CBS) and 15.5 dNm (MBTS). During compounding, high shear forces are generated, which favors rubber reclaiming. The presence of some chemical agents may enhance the effect [30]. If an applied agent can create free radicals and has an affinity to the sulfur, as do typical commercially available rubber vulcanization accelerators, they produce a more effective reclaiming and hence, better processing; for example, tetramethyl thiuram disulfide (TMTD) or even CBS or MBTS. This is reflected in the presented results, where the lowest values were obtained for the mentioned accelerators, while the highest was observed for sulfur. The small difference between S and S80 may result from the fact that S80 is dispersed in an elastomeric binder. Regarding GTR/TOR^SDT^, the minimal torque is rather high compared to CBS and MBTS, even though it is a commercially used accelerator and reacts as a sulfur donor. It may be that during the sample preparation the revulcanization process partially occurred. Those assumptions are supported with the scorch time and optimum cure time results, which are the lowest for the SDT sample (1.1 and 3.4 min, respectively). 

The maximal torque (M_max_.) value, which translates to the stiffness of a material, differs significantly depending on the accelerator type and decreases as follows: 44.5 dNm (S), 41.6 dNm (S80), 34.8 dNm (SDT), 29.7 dNm (CBS) and 27.9 dNm (MBTS). The same trend is noticeable for torque increment (ΔM) (25.3 dNm, 22.9 dNm, 15.9 dNm, 13.8 dNm, and 12.4 dNm for S, S80, SDT, CBS, and MBTS, respectively. This shows how efficient, in the case of revulcanization, is every particular component. 

Samples cured with sulfur (GTR/TOR^S^ and GTR/TOR^S80^) show scorch and optimum cure times similar to each other (1.5 and 4.6 min scorch and 4.2 min optimum cure time, respectively). For samples cured with sulfur, the inversion was noticed. This means that after reaching the alleged maximum torque, set by the software, additional reactions took place. This phenomenon has been confirmed using the DSC method and commented later in the manuscript.

In the case of the accelerators applied, the scorch time was 2.3, 2.2 and 1.1 min and the optimum cure time was 4.5, 6.3, and 3.4 min, respectively, for GTR/TOR^CBS^, GTR/TOR^MBTS^, and GTR/TOR^SDT^. In general, the vulcanization of rubber only with a help of an accelerator is rather limited in the absence of sulfur. However, it can happen when a high temperature, close to the decomposition temperature of the accelerator, is applied. The processing of GTR, especially when it is not highly reclaimed, is limited and requires high temperature and high pressure, which favors revulcanization solely with the presence of an accelerator. The difference between the obtained results is mainly due to the type of decomposition products and their vulcanization mechanics e.g., CBS decomposes under high temperature (205 °C) to 2-bisbenzothiazole-2,2′-disulfid (MBTS), 2-bisbenzothiazole-2,2′-polysulfides (MBTP), 2-bisbenzothiazole-2,2′-monosulfide (MBTM), 2-mercaptobenzothiazole (MBT), and N-cyclohexylamino-2-benzothiazol polysulfides (CBP) and 2-N-cyclohexylaminobenzothiazole (CB) [31]. Upon prolonged heating, only MBT and CB remain. Even though the revulcanization process takes place at 180 °C (which is lower compared to the decomposition temperature of CBS), high pressure is applied, which compensates for the temperature difference resulting in the creation of the aforementioned CBS decomposition products. The difference in the type of the products shifts the time required to obtain a material with optimal properties, which is visible when the optimum curing time of GTR/TOR^CBS^ is compared to GTR/TOR^MBTS^ (4.5 and 6.3 min, respectively).

The parameter R300 for the tested samples was 0.7, 2.1, 0.5, 0.9, and −3.5% for GTR/TOR^S^, GTR/TOR^CBS^, GTR/TOR^MBTS^, GTR/TOR^SDT^, and GTR/TOR^S80^, respectively. The parameter was not determined for GTR and GTR/TOR due to the fact that for these samples the cross-linking efficiency cannot be measured by an oscillating disc rheometer. The negative value for GTR/TOR^S80^ is due to the fact that the sample additionally cross-links after reaching maximum torque; this is explained in the further parts of the manuscript (DSC).

### 3.2. FTIR Analysis

**Figure 3:**
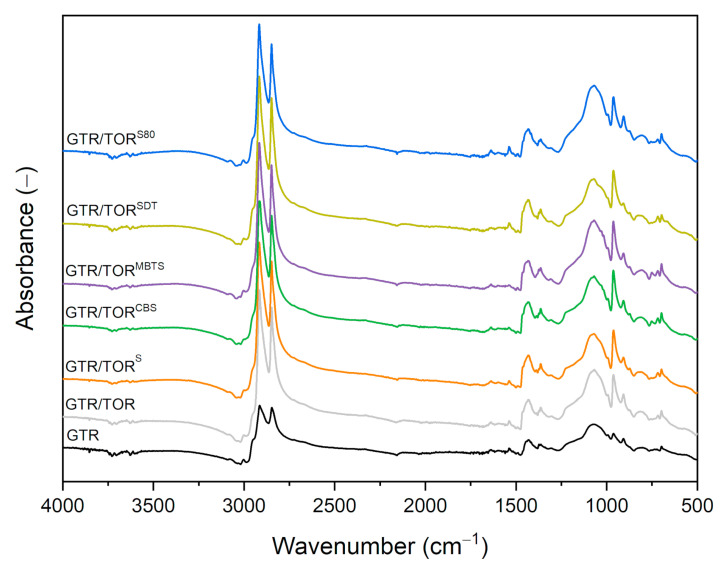
FTIR spectra of the studied compounds.

The FTIR spectra of studied samples are presented in Figure 3. The analysis of obtained results shows that there are no significant differences between specimens. The bands of C–H bonds of CH_2_ groups existing in the aliphatic chains of elastomers are located at 2915 cm^−1^ and 2850 cm^−1^. The peak at approx. 1437 cm^−1^ is associated with C–H bonds of –C=CH_2_, while the band at approx. 1367 cm^−1^ can be connected with C–H bonding of the –CH_3_ groups. As can be noticed, the intensity of those two bands is rising with the presence of TOR, which is rather an obvious output due to its structure. The same phenomenon occurs also for band 965 cm^−1^, which corresponds to a trans-C–H out-of-plane bend. Another interesting band that appears due to the presence of TOR in the GTR/TOR compounds is at 700 cm^−1^ that is assigned to the cis-C–H out-of-plane bend. The band at 807 cm^−1^ corresponds to the skeletal vibration of C–C bonds. In the range of 1100 cm^−1^ to 880 cm^−1^, C–O–C bonding as well as S=O, C–C, and C–O bonds are present, which are detected due to the structure of applied components and their transformation (oxidation of GTR, revulcanization etc.). The presented results indicate that the chemical structure of modified GTR has not changed regardless of the presence of TOR and/or a curing agent, or that these changes are too small to be observed by the FTIR technique.

### 3.3. Physico-Mechanical Properties

**Figure 4:**
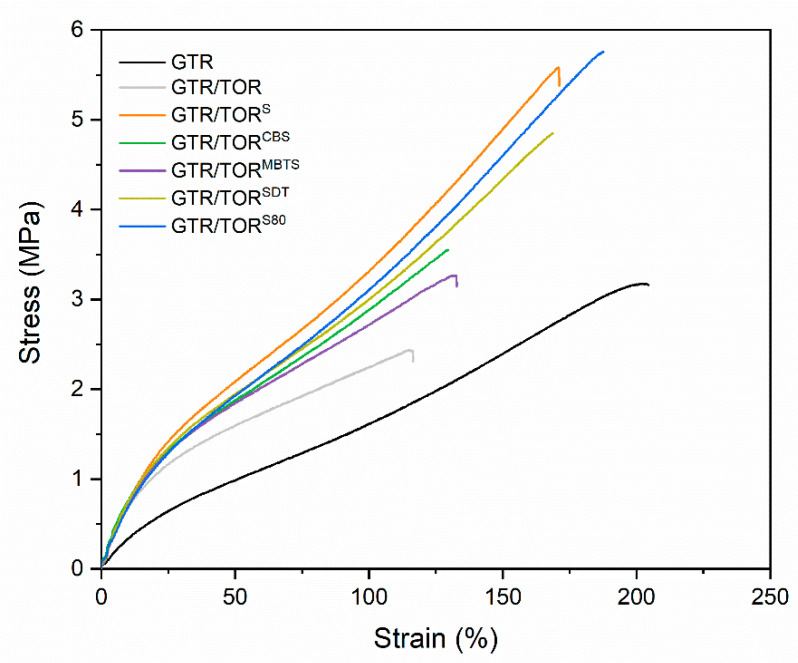
Stress–strain curves of the studied samples.

The physico-mechanical properties of studied samples are presented in Table 4. For a better presentation of the results, the strain–stress curves are shown in Figure 4. Samples without cross-linking additives, coded as GTR and GTR/TOR, were prepared in the same manner as the others, while the curing time was set for 5 min. The results for tensile strength and elongation at break are as follows: 3.1 ± 0.1 MPa (GTR), 2.4 ± 0.2 MPa (GTR/TOR), 5.5 ± 0.3 MPa (GTR/TOR^S^), 3.5 ± 0.3 MPa (GTR/TOR^CBS^), 3.3 ± 0.2 MPa (GTR/TOR^MBTS^), 4.7 ± 0.3 MPa (GTR/TOR^SDT^), 5.8 ± 0.4 MPa (GTR/TOR^S80^) MPa and 198 ± 3% (GTR), 111 ± 13% (GTR/TOR), 167 ± 8% (GTR/TOR^S^), 128 ± 14% (GTR/TOR^CBS^), 132 ± 11% (GTR/TOR^MBTS^), 164 ± 8% (GTR/TOR^SDT^) and 186 ± 5% (GTR/TOR^S80^). The addition of TOR caused a drastic drop in tensile strength and elongation at break values for approx. 23% and 44% compared to unmodified GTR. This shows that the addition of TOR, without any chemical treatment, has a negative impact on the final tensile properties. The absence of any curing agent prevents chemical reclaiming, cross-linking of TOR, and any co-cross-linking between GTR and TOR. The presence of the uncured component disrupts the sintering process of GTR, so the properties of GTR/TOR are the combined results of the hampered sintering process and uncured TOR (proved by the increase of sol fraction), which has resulted in a lower tensile strength and elongation at break than those of GTR. The possibility of a lack of compatibility with the matrix was ruled out by SEM analysis (see subchapter 3.5). The change of M100 (1.6 ± 0.1 to 2.2 ± 0.1 MPa), hardness (56 ± 1 to 68 ± 1 Shore A), density (1.178 ± 0.002 to 1.145 ± 0.008 g/cm^3^), swelling degree (163.5 ± 3.2 to 172.1 ± 4.2%), and sol fraction (10.5 ± 0.1 to 20.5 ± 0.5%), with the addition of TOR, results from the characteristics of the modifier. 

Among the studied materials, there are two samples cross-linked with sulfur, however, one of them is dispersed in ethylene–propylene–diene monomer rubber (EPDM) (80% sulfur). The physico-mechanical characteristics of GTR/TOR^S^ and GTR/TOR^S80^ are different, and the specific parameters are: 5.5 ± 0.3 and 5.8 ± 0.4 MPa (tensile strength), 167 ± 15 and 186 ± 19% (elongation at break), 72 ± 1 and 72 ± 1 Shore A (hardness), 1.164 ± 0.008 and 1.153 ± 0.002 g/cm^3^ (density), and 138.9 ± 8.7 and 149.3 ± 0.4% (swelling degree) and 9.8 ± 0.4 and 10.4 ± 0.1% (sol fraction), respectively for GTR/TOR^S^ and GTR/TOR^S80^. For the S80 curing agent, EPDM acts as a physical binder, which facilitates the access of sulfur to the unsaturated bonds and free radicals obtained during the sintering process. However, as it was confirmed by the inversion phenomenon (curing characteristics) and DSC analysis, the presented properties apply to samples that are not fully cross-linked.

A similar analysis was carried out for samples cross-linked with CBS, MBTS, and SDT accelerators, showing that SDT has the highest influence on the physico-mechanical properties of the studied specimens. It provided the highest tensile strength (4.7 ± 0.3 MPa), stiffness (3.0 ± 0.1 MPa), hardness (71 ± 1 Shore A), and density (1.141 ± 0.001 g/cm^3^) compared to GTR/TOR^CBS^ and GTR/TOR^MBTS^. 

The sol fraction increases as follows: GTR/TOR^S^ (9.8 ± 0.4%), GTR/TOR^S80^ (10.4 ± 0.1%), GTR (10.5 ± 0.1%), GTR/TOR^SDT^ (12.5 ± 0.1%), GTR/TOR^CBS^ (15.9 ± 0.3%), GTR/TOR^MBTS^ (17.9 ± 0.1%) and GTR/TOR (20.5 ± 0.5%). GTR/TOR^S^, GTR/TOR^S80^, and GTR have similar values indicating the mentioned sulfur cross-linking of GTR and TOR. Among the applied accelerators, the lowest sol fraction was obtained by SDT, which is in accordance with the physico-mechanical properties. MBTS and CBS have similar cross-linking characteristics, however, the sol fraction of GTR/TOR^MBTS^ is higher. This phenomenon can be related to partial devulcanization of GTR produced by the used accelerators, which corresponded with a lower value of minimal torque measured during the study of the curing characteristics. 

The basic physico-mechanical properties (tensile strength, elongation at break and hardness) of the studied samples were compared with the results reported by different research groups and our previous studies and are shown in Table 5. The table consists of materials, whose main component is GTR (similar to the present study) and materials with much lower content of waste rubber. It can be seen that the designed systems meet or even exceed results published by independent research groups. In case of samples with similar GTR content (GTR/recycled PE/TOR 90/10/9, GTR/EVA 100/10 and GTR/Bitumen/PCL 90/10/10), materials presented in this study (GTR/TOR/active compound 90/10/3) are characterized with higher tensile strength (~2.1, 2.7–3.4, 2.1–2.4 and 3.1–5.8 MPa, respectively) and elongation at break (~70, 125–164, 89–92 and 128–198%, respectively). Even for the material in which the amount of GTR was reduced more than twice and dynamically vulcanized with a complete sulfur curing system in the presence of TOR (GTR/PP/TOR/curing system 60/40/10/9), the obtained results are characterized with higher tensile strength (137% of GTR/TOR^S80^) and significantly reduced elongation at break (13.4% of GTR/TOR^S80^). Considering the significant reduction of waste material and the significant decrease of elongation at break, the materials presented in this article show satisficing performance properties.

### 3.4. Thermogravimetric Analysis

**Figure 5:**
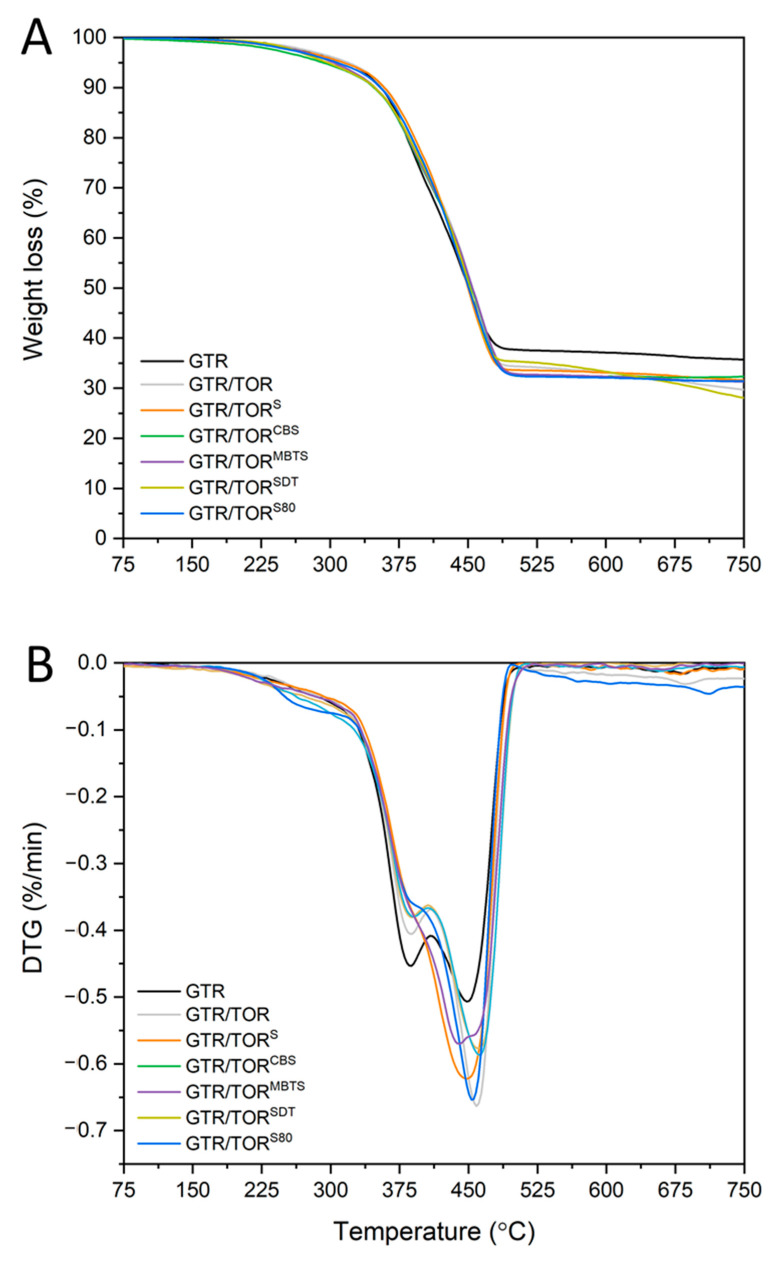
(**A**) TGA and (**B**) DTG curves of the studied samples.

The results of the thermogravimetric analysis of GTR, GTR/TOR, GTR/TOR^S^, GTR/TOR^CBS^, GTR/TOR^MBTS^, GTR/TOR^SDT,^ and GTR/TOR^S80^ are presented in Figure 5 and summarized in Table 6. As can be seen, the addition of TOR shifts the thermal stability of GTR/TOR (compared to GTR) towards higher values (T_−2%_—258.0 to 261.2 °C and T_−5%_ 315.5 to 318.7 °C). This is due to the good thermal stability of TOR—275 °C. The scale of changes is small, which is a result of the amount of the additive used. The rest of the curing agents caused a shift of thermal stability towards lower values compared to GTR/TOR sample (T_−2%_ −261.2. 245.9. 225.9. 253.2. 250.6 and 243.1 °C along with T_−5%_—318.7, 313.4, 290.9, 303.2, 295.6 and 305.6 °C, respectively for GTR/TOR, GTR/TOR^S^, GTR/TOR^CBS^, GTR/TOR^MBTS^, GTR/TOR^SDT^, and GTR/TOR^S80^). The thermal stability of rubber increases with an increasing degree of cross-linking. However, in this case, the opposite phenomenon takes place. The presence of any cross-linkers and accelerators may decrease the thermal stability of rubber products [37]. These components can be thermally decomposed by the effect of elevated temperatures, forming free radicals. Such decomposition products have a significant ability to initiate reactions involving main chains and network nodes affecting the thermal stability of the material. The same phenomenon is commonly used to carry out processes of thermo-chemical reclaiming of GTR. 

The aforementioned accelerated decomposition also affects DTG (derivative thermogravimetry) curves (Figure 5B). The change of intensity and the shift of two characteristic decomposition peaks of GTR (approx. 390 °C—natural rubber and approx. 445 °C—styrene-butadiene rubber [37]) can be a result of cross-linking and co-cross-linking between the rubber matrix and the TOR, as well as reclaiming. The content of char residues read at 750 °C varies up to 30% for all samples containing TOR. Changes of 2% to 4% cannot be very significant if a sample of about 10 mg is analyzed. The high value of char in GTR is reasonable due to the presence of carbon black, SiO_2_ and inorganic impurities. 

### 3.5. Scanning Electron Microscopy

**Figure 6:**
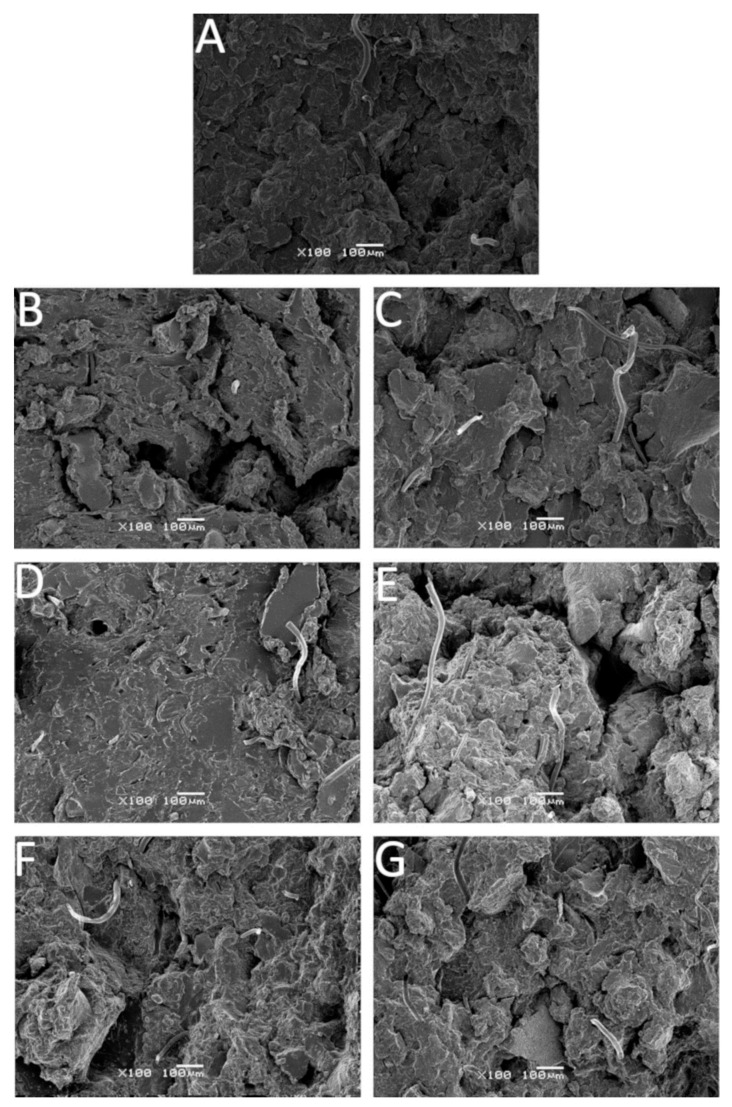
SEM images of samples: (**A**)—GTR; (**B**)—GTR/TOR; (**C**)—GTR/TOR^S^; (**D**)—GTR/TOR^CBS^; (**E**)—GTR/TOR^MBTS^; (**F**)—GTR/TOR^SDT^; (**G**)—GTR/TOR^S80^ (magnification ×100). Abbreviations: GTR—ground tire rubber; TOR—trans-polyoctenamer rubber.

The impact of TOR and applied chemical modifiers on the morphology of the studied samples is presented in Figure 6. SEM images show the surface area perpendicular to the direction of strain, which was created by the breaking of the samples subjected to a static tensile test (the cross-head speed was 500 mm/min). As can be seen, all of the studied samples are characterized by a rather rough surface, however, a few distinctive features can be noticed. Figure 6A represents the GTR sample, which reveals outgoing fibers (residues from tire reinforcements) as well as the voids and gaps created by the rubber particles being torn out during the tensile test. For the GTR/TOR (Figure 6B), the same observations were made, except for the increase in surface roughness. This change was caused by the addition of TOR, which is compatible with GTR. Despite this compatibility, the mechanical properties of the sample are lower than those of the reference. The forces acting on the specimen have been partially transferred to the TOR. The mentioned additive was not cross-linked due to the lack of curing agents and it could not carry the applied load. In the case of pure GTR, the material was sintered and there was no TOR hindering the process, which resulting in superior mechanical properties. 

The sample cured with sulfur (Figure 6C) is very similar to the reference sample with visible waste rubber particles, while sulfur dispersed in ethylene–propylene–diene monomer rubber (EPDM) (Figure 6G) is characterized by a rough and shredded surface, which indicates a more developed structure. As mentioned before, the EPDM may act as an additional binder facilitating the cross-linking process by reactive sintering. 

The sample treated with CBS (Figure 6D) is relatively smooth with few voids and gaps. The presence of CBS supports the process of reactive sintering and the tested area is the most homogenous among the tested samples. The surface of GTR/TOR^SDT^ (Figure 6F) has a well-developed surface and this is reflected in its mechano-physical properties. GTR/TOR^MBTS^ (Figure 6E) also shows good surface development, however, its properties are not as good as those of GTR/TOR^SDT^. 

The results show that the type of accelerator has an influence on TOR dispersion between rubber particles, but the most important factor is the curing mechanism, which depends on the type of curing agent. Moreover, the smooth surface, which is commonly associated with the appropriate dispersion of components and their good compatibility, does not correlate completely in the presented study. Only a properly developed surface, increasing the possibility of reactive sintering and the creation of a higher number of cross-links, combined with a properly selected cross-linking system, allows obtaining a product with satisfactory parameters. Due to the cross-linked structure it is rather hard to properly develop a convenient surface of GTR. However, the application of TOR, which is compatible with GTR, makes this possible. 

### 3.6. Acoustic Properties

**Figure 7:**
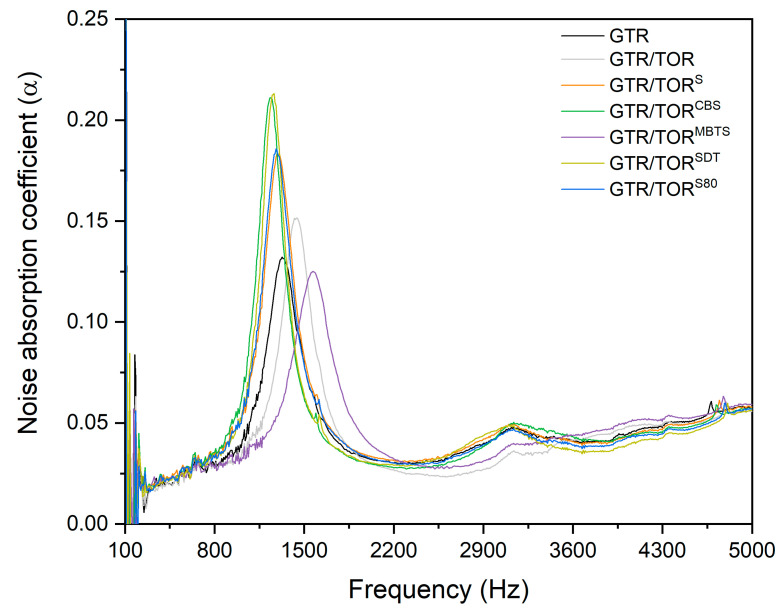
Sound absorption coefficient as a function of frequency.

The sound absorption coefficient as a function of the frequency of the studied samples is presented in Figure 7. For all of the tested specimens, a significant peak was recorded at the frequency near 1000–1500 Hz. The acoustic properties depend on the chemical and physical structure of the tested material and they are strongly related to the material’s density [38]. Therefore, the acoustic properties result directly from the type of material, particle size distribution, methods of its preparation and thickness [39]. The peak in the 1000 to 1500 Hz range is common for GTR, which has been confirmed by independent studies [38,39,40,41], and it can be treated as a material characteristic. Depending on the applied curing system, the intensity of the mentioned peak changes. Additionally, a small band is visible for GTR/TOR, GTR/TOR^S^, GTR/TOR^CBS^, GTR/TOR^SDT^ and GTR/TOR^S80^ at 2600 to 3500 Hz. 

The sound absorption is related to sample structure, properties, thickness, and surface conditions, as well as to the incident angle and frequency of the sound waves [40]. The measured value changes as the frequency changes from low, through the middle, and up to the high values. In that case, to evaluate the sound-absorbing property one can determine the noise absorption coefficient for specific frequency values (125, 250, 500, 1000, 2000, and 4000 Hz). When the average frequency equals or is higher than 0.2, the tested material can be called “sound-absorbing”. In Table 7, the specific values and the average for every tested sample are presented. The results show, that none of the tested specimens fulfills the requirements. The highest value was recorded for GTR/TOR (0.042). However, the applied method misses the frequencies at which tested materials show higher sound absorption. To evaluate the method, another one was used according to the literature studies [41].

Table 8 shows the average sound absorption coefficient at low (100–315 Hz), medium (400–1250 Hz) and high (1600–4000 Hz) frequencies. These values are higher than those presented in Table 6, however, none of them meet the expected requirements. 

### 3.7. Differential Scanning Calorimetry 

**Figure 8:**
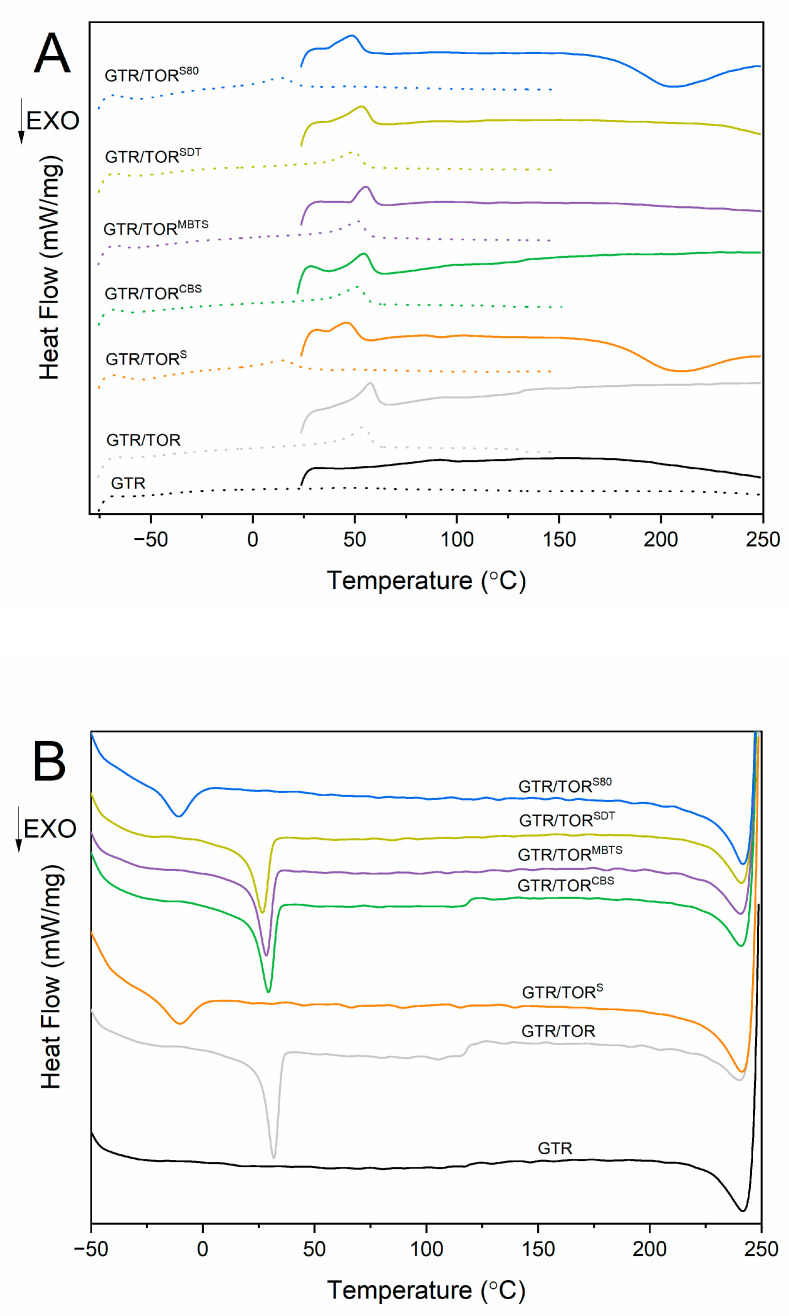
Differential scanning calorimetry (DSC) thermogram evaluating the melting (**A**) and crystallization (**B**) of the studied samples (straight line—1st run; dot line—2nd run).

To evaluate the influence of curing additives on the co-cross-linking of TOR and GTR, a DSC study was conducted. The thermogram representing the heating (Figure 8A) and cooling (Figure 8B) process of the revulcanizates is presented in Figure 8. Moreover, enthalpy (ΔH), initial temperature (T_ω_), peak temperature (T_p_), temperature range (D), and time (t) of the melting and crystallization process are presented in Table 9. For better understanding, pure TOR was analyzed.

The results show that the only (except the samples cured with sulfur) melting/crystallization peak recorded by the method is associated with TOR. As is well known, the first heating run is conducted to remove the thermal history of a sample, changing the enthalpy and crucial temperatures compared to the second heating run. In the case of GTR/TOR, GTR/TOR^CBS^, GTR/TOR^MBTS^, and GTR/TOR^SDT^ the enthalpy changes from 5.76, 4.86, 3.32, and 4.88 J/g to 5.06, 4.35, 4.13, and 3.87 J/g, respectively, while peak temperature changes from 57.6, 54.3, 55.4, and 53.2 J/g to 54.3, 51.1, 51.3, and 49.4 J/g, respectively. 

The most interesting results were noticed for samples cured with sulfurs. For GTR/TOR^S^ and GTR/TOR^S80^ two peaks were recorded: first at 45.9 and 48.4 °C (associated with the melting of TOR), while the second at 210.4 and 205.7 °C, respectively. The second peak was only visible during the first heating run. This phenomenon is associated with the previously discussed, in subchapter 3.1, occurrence and it is correlated with undesirable reactions during DSC analysis. As mentioned before, the cross-linking characteristics indicated that, with the ongoing measurement, the sample does not reach the plateau, and inversion is observed (Figure 2). It was found that the applied conditions were not sufficient to obtain optimum parameters. This is due to the fact that in standard rubber compounds accelerators and activators are used for the cross-linking process with sulfur. The lack of the mentioned additives significantly prolongs this process, which can be observed in the presented paper. The phenomenon was also confirmed by the 2nd heating, which shifts the melting peak to the lower values (45.9 to 14.1 °C and 48.4 to 13.4 °C, respectively for GTR/TOR^S^ and GTR/TOR^S80^) and decreases the ΔH_m_ values. It was also noticed for the crystallization behavior, where the initial temperature shifted from 39.9 °C (TOR) to 5.3 and 3.8 °C. The D_m_ parameter of the 1st heating for the second peak was 72.4 and 82.3 °C, respectively for GTR/TOR^S^ and GTR/TOR^S80^. This shows that the reactions ended approx. at 230 to 240 °C. This exothermic reaction is a vulcanization process. These findings are in accordance with independent studies [42].

## 4. Conclusions

The proposed method of GTR utilization involves the application of a commercially available waste rubber modifier—TOR (Vestenamer 8012). The idea was to investigate the influence of curing agents/accelerators on curing characteristics, physico-mechanical, thermal, acoustic properties and morphology of the modified GTR. As reference samples, a pure GTR and GTR modified by TOR without any chemical treatment were used. All samples were reclaimed using a two-roll mill and then reactively sintered according to the optimum curing time. 

The results show that it is possible to cross-link GTR using only curing additives, without the addition of auxiliary components (plasticizers, activators, etc.). Moreover, curing characteristics depend on the type of compound used, i.e., the most important influence is the nature of the reaction that this compound generates. During the study, GTR/TOR^S^ and GTR/TOR^S80^ showed an inversion phenomenon indicating the appearance of additional chemical reactions after reaching the maximum of vulcanization. This indicates that sulfur can revulcanize GTR, however without accelerators, activators, and plasticizers, the process is unstable. 

The physico-mechanical analysis showed a deterioration of GTR/TOR properties compared to pure GTR. This indicates that TOR does not create a cross-link with GTR without additional chemical treatment. Moreover, the presence of TOR hinders the sintering process between GTR particles, unless a curing agent/accelerator is introduced into the rubber mix. The best physico-mechanical properties were obtained by samples cured with sulfurs (5.5 ± 0.3 and 5.8 ± 0.4 MPa—tensile strength, 167 ± 15 and 186 ± 19%—elongation at break, 72 ± 1 and 72 ± 1 Shore A—hardness, respectively GTR/TOR^S^ and GTR/TOR^S80^). Among the applied accelerators the best properties were obtained for GTR/TOR^SDT^ (4.7 ± 0.3 MPa—tensile strength, 164 ± 8%—elongation at break, 71 ± 1 Sh A—hardness, density g/cm^3^—1.141 ± 0.002. 

The analysis of the morphology of the tested specimens shows that chemical modifiers influence TOR dispersion as well as the curing mechanism. In the case of the sulfur cured samples, the presence of EPDM in S80 additive influences the development of the GTR particles. 

During the DSC tests, the appearance of an exothermic reaction resulting from the presence of sulfur in the composition was observed. The results indicate that the selected process conditions which were sufficient for the vulcanization by accelerators were not sufficient to obtain a product with optimal parameters using only sulfur. Nevertheless, samples treated with accelerators showed decreases in melting enthalpy of TOR compared to GTR/TOR, indicating enhanced TOR degradation and/or co-cross-linking between GTR and the modifier.

The acoustic properties showed that the materials cannot be used as sound-insulating products. However, the proposed method of reclaiming and modification of GTR may allow introducing the tested material into other polymer matrices (polyurethane foams or foamed rotational moulding products), improving their acoustic properties, reducing the cost of production, influencing the ecological aspect of the product, and broadening the range of potential applications. 

The presented results indicate that the obtained materials are characterized by good performance properties, compared to the results presented by other research groups (when GTR is considered as the main component of the product). The obtained parameters are sufficient to utilize the recycled GTR as a material for the production of anti-vibration mats, dustbin wheels, water-proof rubber goods and roofing elements (the conclusion drawn after discussions with manufacturers). As part of further work, tests of thermal conductivity and water absorption are planned to determine the susceptibility of the material to the above mentioned purposes.

## Figures and Tables

**Table 1:** Characteristics of trans-polyoctenamer rubber (Vestenamer 8012) *.

Property	Mooney Viscosity ML(1 + 4) at 100 °C (MU)	Glass Transition Temperature (°C)	Melting Point (°C)	Crystallinity (%)	Thermal Degradation (°C)	Tensile Strength (MPa)	Elongation at Break (%)
Method	DIN 53 523	ISO 11357-1/-2	ISO 11357-1/-3	DSC (2nd heating)	TGA	ISO 527	ISO 527
Value	<10	−65	54	~30	275	8.5	400

* Information according to datasheet from the producer.

**Table 2:** The composition and coding of studied samples.

Components (phr)	Sample Code						
	GTR	GTR/TOR	GTR/TOR^S^	GTR/TOR^CBS^	GTR/TOR^MBTS^	GTR/TOR^SDT^	GTR/TOR^S80^
GTR	100	90	90	90	90	90	90
TOR	-	10	10	10	10	10	10
Sulfur	-	-	3	-	-	-	-
CBS	-	-	-	3	-	-	-
MBTS-80	-	-	-	-	3.75	-	-
SDT-50	-	-	-	-	-	6	-
S-80	-	-	-	-	-	-	3.75

**Table 3:** Curing characteristics of studied samples performed at 180 °C.

Properties	GTR	GTR/TOR	GTR/TOR^S^	GTR/TOR^CBS^	GTR/TOR^MBTS^	GTR/TOR^SDT^	GTR/TOR^S80^
Minimal torque (dNm)	-	-	19.2	15.8	15.5	18.9	18.7
Maximal torque (dNm)	-	-	44.5	29.7	27.9	34.8	41.6
ΔM (dNm)	-	-	25.3	13.9	12.4	15.9	22.9
Scorch time (min)	-	-	1.5	2.3	2.2	1.1	1.5
Optimum cure time (min)	5.0	5.0	4.6	4.5	6.3	3.4	4.2
Cure rate index (min^−1^)	-	-	31.9	44.6	24	42.7	38
Thermal aging resistance (%)	-	-	0.7	2.1	0.5	0.9	−3.5

**Table 4:** Physico-mechanical properties of the studied samples.

Properties	GTR	GTR/TOR	GTR/TOR^S^	GTR/TOR^CBS^	GTR/TOR^MBTS^	GTR/TOR^SDT^	GTR/TOR^S80^
Tensile strength (MPa)	3.1 ± 0.1	2.4 ± 0.2	5.5 ± 0.3	3.5 ± 0.3	3.3 ± 0.2	4.7 ± 0.3	5.8 ± 0.4
Elongation at break (%)	198 ± 3	111 ± 13	167 ± 8	128 ± 14	132 ± 11	164 ± 8	186 ± 5
M100 (MPa)	1.6 ± 0.1	2.2 ± 0.1	3.3 ± 0.1	2.8 ± 0.1	2.7 ± 0.1	3.0 ± 0.1	3.2 ± 0.1
Hardness (Shore A)	56 ± 1	68 ± 1	72 ± 1	72 ± 1	70 ± 1	71 ± 1	72 ± 1
Density (g/cm^3^)	1.178 ± 0.002	1.145 ± 0.008	1.164 ± 0.001	1.146 ± 0.003	1.150 ± 0.008	1.141 ± 0.002	1.153 ± 0.002
Swelling degree (%)	163.5 ± 3.2	172.1 ± 4.2	138.9 ± 8.7	170.7 ± 1.6	176.4 ± 1.3	159.8 ± 0.4	149.3 ± 0.4
Sol fraction (%)	10.5 ± 0.1	20.5 ± 0.5	9.8 ± 0.4	15.9 ± 0.3	17.9 ± 0.1	12.5 ± 0.1	10.4 ± 0.1

**Table 5:** Physico-mechanical properties of GTR-based blends reported by different groups and previous studies.

Sample Composition	Sample Preparation	Tensile Strength (MPa)	Elongation at Break (%)	Hardness (Shore A)	Ref.
GTR/TOR/active compound 90/10/3	mixing at ambient temperature compression molding 180 °C	3.1–5.8	128–198	56–72	This study
GTR/recycled PE/TOR 90/10/9	extrusion at 150–180 °C injection molding at 180–190 °C	~2.1 *	~70 *	~78 *	[22]
GTR/bitumen/PCL 90/10/10	mixing GTR with bitumen at ambient temperature, then mixing with PCL at 120 °C compression molding at 120 °C	2.1–2.4	89–92	61–63	[32]
GTR/EVA 100/10	extrusion at 60 °C, compression molding at 140–180 °C	2.7–3.4	125–164	63–65	[33]
waste thermoplastic polyurethane/waste SBR/PE-*g*-MA 90/10/5	mixing at 170–175 °C compression molding at 170 °C	4.8	280	81	[34]
recycled LDPE/GTR/EVA 30/40/30	extrusion at 165–175 °C injection molding at 165–190 °C	~7.8 *	~180 *	unknown	[35]
GTR/PP/TOR/curing system 60/40/10/9.5	mixing at 180 °C (dynamic vulcanization)	~8.0	~25	unknown	[36]

* the value estimated from graphs.

**Table 6:** Thermal decomposition characteristics of tested samples estimated from TGA data.

Sample	T_−2%_	T_−5%_	T_−10%_	T_−50%_	Char Residues at 750 °C
GTR	258.0	315.5	355.5	450.5	35.7
GTR/TOR	261.2	318.7	356.2	453.7	29.7
GTR/TOR^S^	245.9	313.4	358.4	448.4	31.6
GTR/TOR^CBS^	225.9	290.9	345.9	453.4	32.3
GTR/TOR^MBTS^	253.2	303.2	348.2	453.2	31.3
GTR/TOR^SDT^	250.6	295.6	348.1	450.6	28.1
GTR/TOR^S80^	243.1	305.6	353.1	450.6	31.4

**Table 7:** The changes in the sound absorption coefficient for the studied samples at 125, 250, 500, 1000, 2000, and 4000 Hz.

Frequency (Hz)	Sample Code						
	GTR	GTR/TOR	GTR/TOR^S^	GTR/TOR^CBS^	GTR/TOR^MBTS^	GTR/TOR^SDT^	GTR/TOR^S80^
	Sound Absorption Coefficient (α)						
125	−0.07661	0.10734	0.03289	0.0307	−0.07653	0.02072	−0.07766
250	0.01146	0.01599	0.01566	0.01529	0.01709	0.00817	0.01525
500	0.02254	0.01958	0.02348	0.01994	0.02095	0.02025	0.01888
1000	0.02352	0.02251	0.02832	0.02433	0.02644	0.02423	0.0233
2000	0.02689	0.0224	0.03775	0.02053	0.03622	0.02178	0.0209
4000	0.05472	0.0623	0.05146	0.03849	0.04037	0.03726	0.03814
Average	0.01042	0.041687	0.031593	0.02488	0.010757	0.022068	0.006468

**Table 8:** The changes of the sound absorption coefficient for the studied samples at low, medium and high frequencies.

Frequency (Hz)	Sample Code						
	GTR	GTR/TOR	GTR/TOR^S^	GTR/TOR^CBS^	GTR/TOR^MBTS^	GTR/TOR^SDT^	GTR/TOR^S80^
	Sound Absorption Coefficient (α)						
100–315	0.06323	0.103741	0.055114	0.056852	0.038187	0.087613	0.051914
400–1250	0.024173	0.02308	0.028704	0.023036	0.025176	0.024022	0.022998
1600–4000	0.038194	0.032915	0.058704	0.025429	0.036743	0.026061	0.025634
Average	0.041866	0.053245	0.047507	0.035106	0.033369	0.045899	0.033515

**Table 9:** Results of the thermal transitions of the samples studied by DSC.

**Sample Code**	**Melting Enthalpy ΔH_m_ (J/g)**		**Initial Melting Temperature (Tω_m_) (°C)**		**Peak Temperature (Tp_m_) (°C)**		**Melting Temperature Range D (°C)**		**Melting Time (t_m_) (min)**	
	**1st run**	**2nd run**	**1st run**	**2nd run**	**1st run**	**2nd run**	**1st run**	**2nd run**	**1st run**	**2nd run**
GTR	-	-	-	-	-	-	-	-	-	-
GTR/TOR	5.76	5.06	43.8	39.9	57.6	54.3	19.4	20.4	1.9	2.0
GTR/TOR^S^	3.60; −20.83	2.85	36.5	1.5	45.9; 210.4	14.1	17.3; 72.4	24.4	1.7; 7.2	2.4
GTR/TOR^CBS^	4.86	4.35	39.7	37.5	54.3	51.1	21.9	20.9	2.2	2.1
GTR/TOR^MBTS^	3.32	4.13	47.3	35.4	55.4	51.3	15.3	24.2	1.5	2.4
GTR/TOR^SDT^	4.88	3.97	36.0	35.2	53.2	49.4	25.4	21.4	2.5	2.1
GTR/TOR^S80^	4.30; −25.49	2.70	35.4	−2.4	48.4; 205.7	13.4	20.8; 82.3	27.0	2.1; 8.2	2.7
TOR	85.88	70.79	40.2	25.4	60.1	56.6	36.9	37.6	2.9	3.7
**Sample Code**	**Crystallization Enthalpy ΔH_c_ (J/g)**		**Initial Crystallization Temperature (Tω_c_) (°C)**		**Peak Temperature (Tp_c_) (°C)**		**Crystallization Temperature Range (D) (°C)**		**Crystalization Time (t_c_) (min)**	
GTR	-		-		-		-		-	
GTR/TOR	−4.74		38.4		31.7		19.9		2.0	
GTR/TOR^S^	−2.47		5.3		−10.2		34.0		3.4	
GTR/TOR^CBS^	−4.41		37.5		29.4		22.0		2.2	
GTR/TOR^MBTS^	−4.15		35.3		28.4		19.2		1.9	
GTR/TOR^SDT^	−3.86		35.3		26.7		20.8		2.1	
GTR/TOR^S80^	−2.24		3.8		−10.8		29.1		2.9	
TOR	−79.07		39.9		32.4		33.9		3.4	
